# Supplementary material for: The interactome of intact mitochondria by cross-linking mass spectrometry provides evidence for coexisting respiratory supercomplexes
Source: Mol Cell Proteomics. 2017 Dec 8;17(2):216–32. doi: 10.1074/mcp.RA117.000470 (PMC5795388; doi:10.1074/mcp.RA117.000470)
Supplement: Supplemental Data [file supp_RA117.000470_133922_0_supp_23978_fzffwf.zip › spectra_annotation/mito_spectra_annotation/253-1-1-1-6-1.pdf]

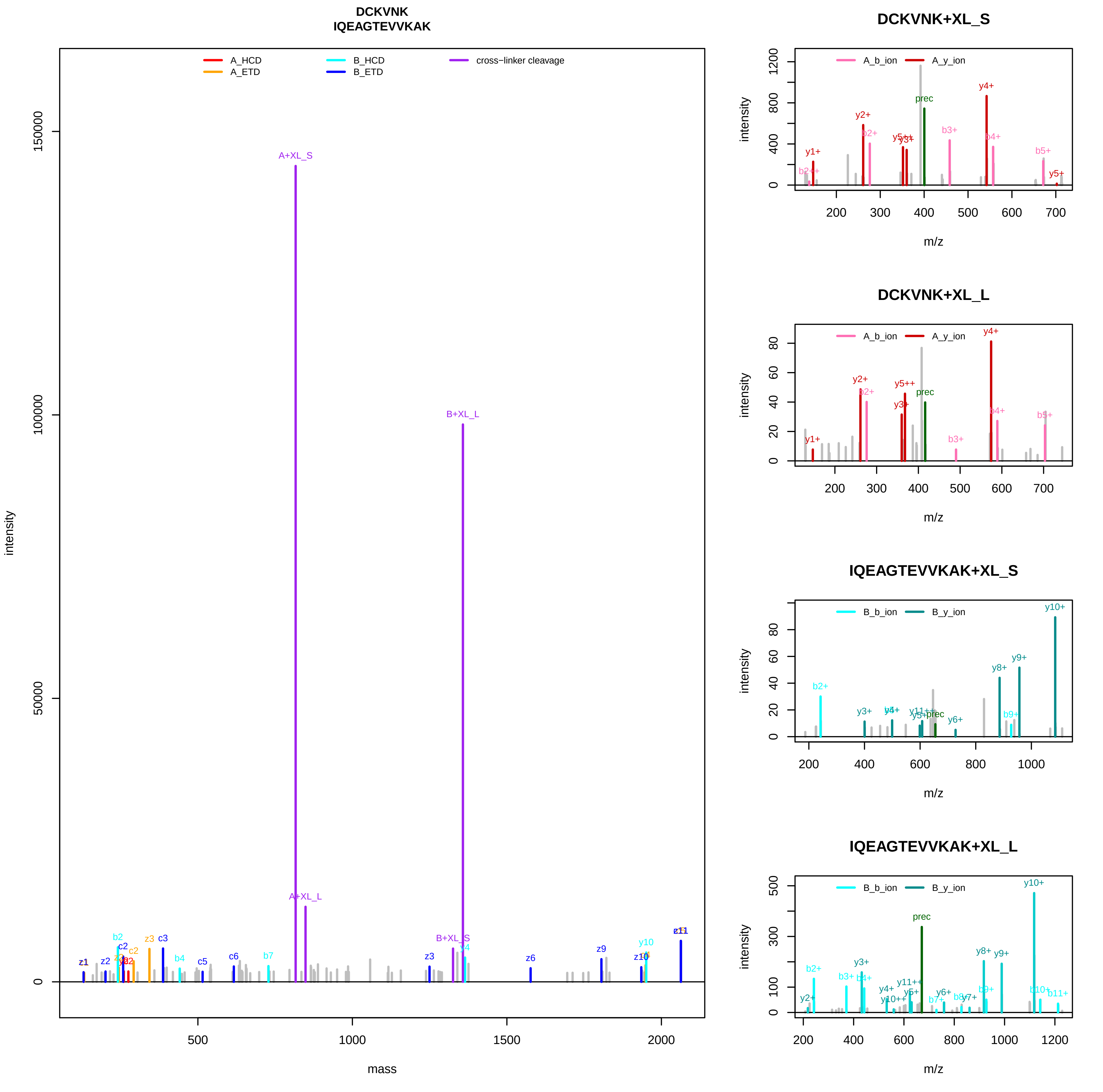
DCKVNK
IQEAGTEVVKAK
A_HCD
A_ETD
B_HCD
B_ETD
cross−linker cleavage
A+XL_S
B+XL_L
A+XL_L
B+XL_S
intensity
mass
0
50000
100000
150000
500
1000
1500
2000
z1
z2
c2
b2
z3
c3
b4
c5
c6
b7
z3
y4
z6
z9
z10
y10
z11
DCKVNK+XL_S
A_b_ion
A_y_ion
y1+
y2+
b2+
y5++
y3+
prec
b3+
y4+
b4+
b5+
y5+
m/z
DCKVNK+XL_L
y4+
y2+
y5++
prec
y3+
b4+
b5+
b3+
y1+
IQEAGTEVVKAK+XL_S
B_b_ion
B_y_ion
y10+
y9+
y8+
b2+
y3+
prec
y6+
b9+
IQEAGTEVVKAK+XL_L
y10+
prec
y8+
y9+
b2+
y3+
b3+
b4+
y4+
y11++
y10++
y5+
y6+
b7+
y7+
b9+
b10+
b11+
y2+
